# Supplementary material for: Neural networks for abstraction and reasoning
Source: Sci Rep. 2024 Nov 13;14:27823. doi: 10.1038/s41598-024-73582-7 (PMC11561310; doi:10.1038/s41598-024-73582-7)
Supplement: Supplementary file 1 — Supplementary Information. [file 41598_2024_73582_MOESM1_ESM.pdf]

Supplementary Material: Neural networks for abstraction and reasoning: Towards broad generalization in machines

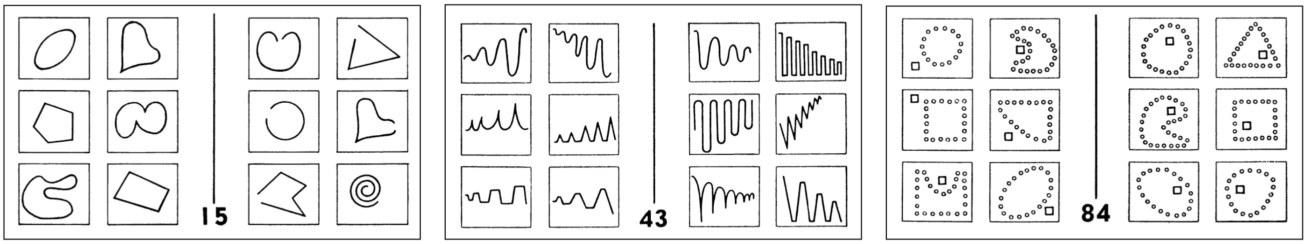

Supplementary Figure 1. Three of Bongard's original problems from 1967. The task is to identify the difference between the two sets, where each problem encapsulates a different concept. **(15)** Set A contains closed shapes while Set B contains open ones. **(43)** Set A contains waves of increasing amplitude; set B decreasing amplitude. **(84)** Set A has a square inside the perimeter defined by connecting the dots; set B outside. Reproduced from [1].

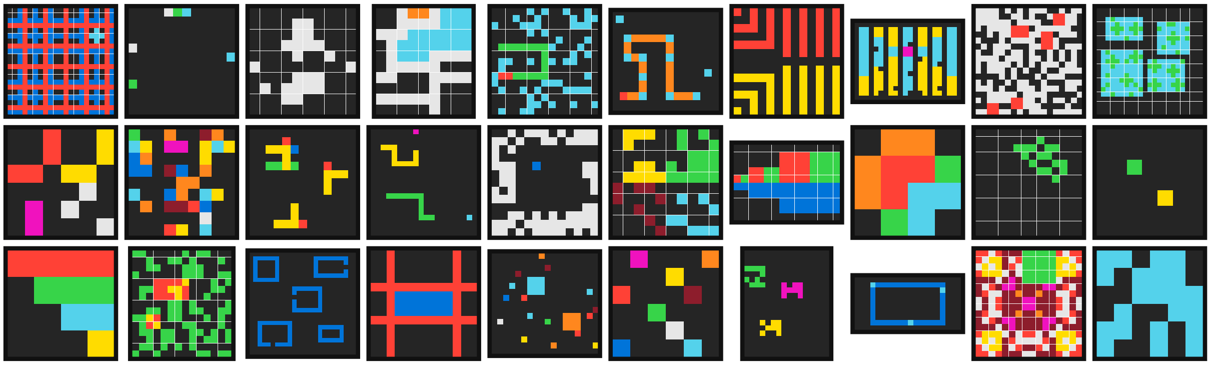

Supplementary Figure 2. The ARC dataset contains 900 handcrafted abstraction and reasoning tasks based on colourful grids. Each task tests new abilities which must be inferred from few examples.

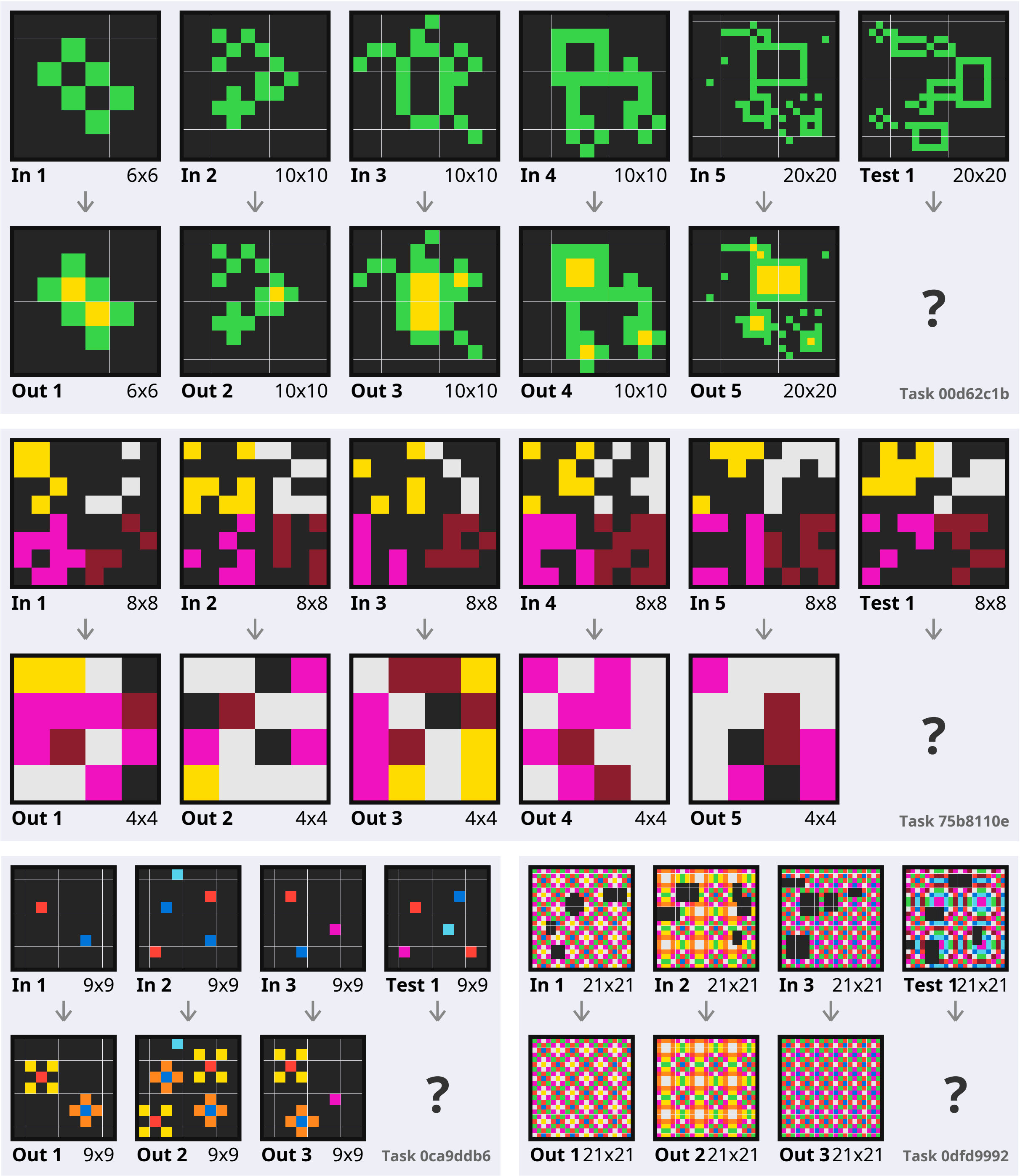

Supplementary Figure 3. Four examples of tasks from the ARC-Easy dataset. Each task requires recognising new patterns and concepts from the training pairs (In/Out) to predict the output of the test examples from the inputs. The tasks have been hand-designed to test a different set of concepts.

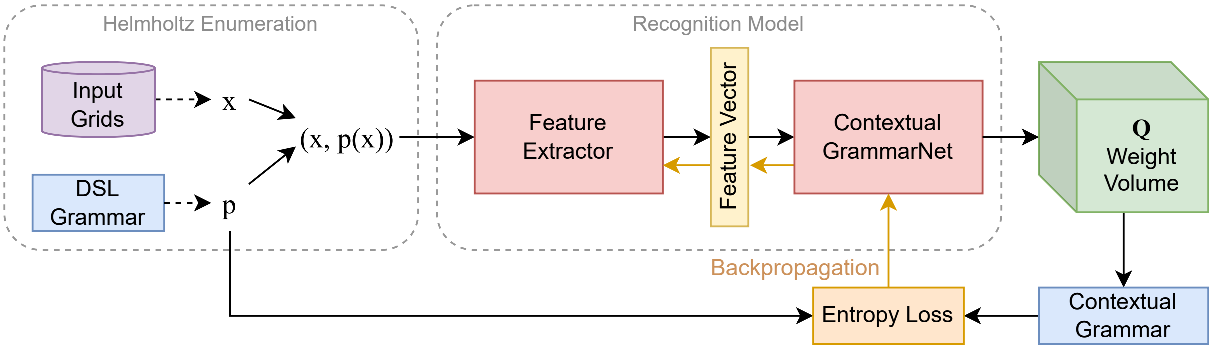

Supplementary Figure 4. The training process for the recognition model. Helmholtz enumeration generates an unlimited stream of dreamed tasks, and the recognition model attempts to produce a contextual grammar which assigns a low entropy to the correct solution for the dreamt tasks. The model outputs a weight volume Q, where $Q_{ijk}(x)$ is the probability of primitive i being the kth argument to primitive j for a given task. Backpropagation optimises the neural network end-to-end.

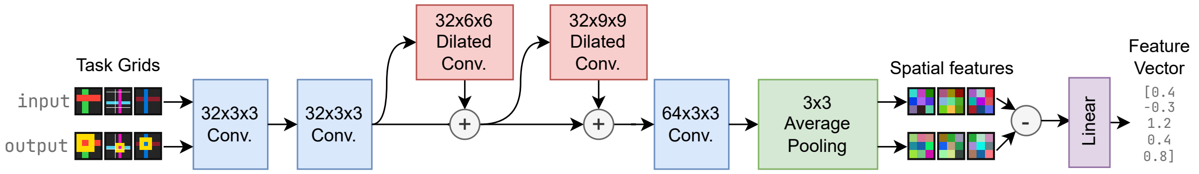

Supplementary Figure 5. The architecture of the fully-convolutional feature extractor. Each grid within an ARC task is operated on by a series of fully-convolutional layers to produce spacial features; the residual between input and output features is turned into a vector that describes the task. The grid size is maintained as long as possible, and the differential of input and output grids are used as features. Residual dilated convolutions ensure multi-scale context can be used. This architecture allows 1x1 up to 30x30 grids to map to the same vector space, as ARC tasks do not have a fixed grid size.

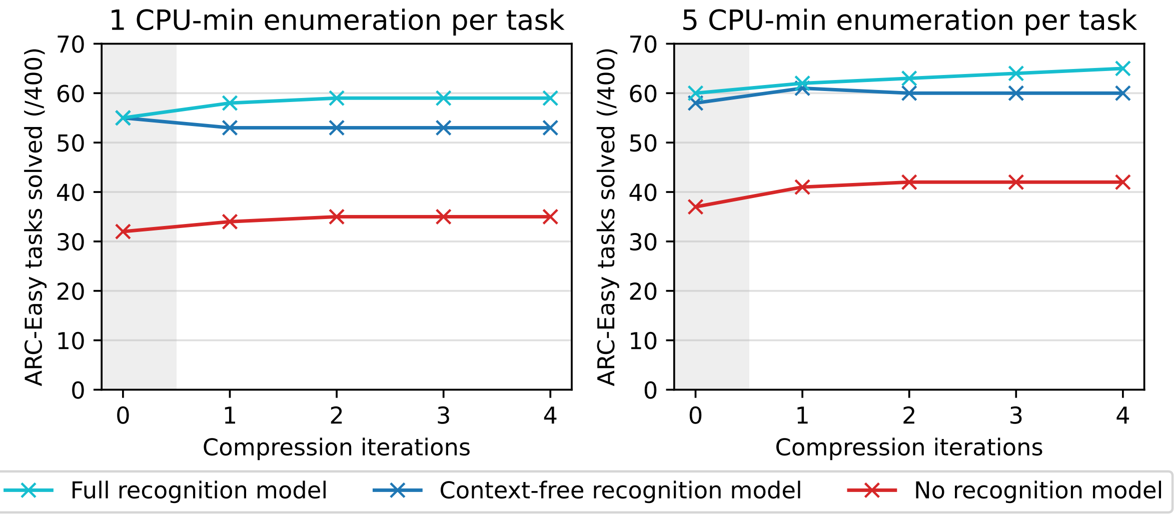

Supplementary Figure 6. The number of ARC-Easy tasks solved by our DreamCoder implementation while ablating two key components. The addition of a neural network recognition model (abstraction sleep) almost doubles the number of solved tasks, while using compression to create new primitives by composing existing ones (dreaming sleep) has a much smaller performance uplift. We also show results with 5x higher enumeration time, showing the effect of a deeper search.

A taxonomy of tasks in ARC

The first step to constructing an ARC solver is to understand the nature of the problem: this helps motivate later design choices. To do this in a systematic way, we built a *taxonomy* of ARC tasks. Some example tasks in this taxonomy are shown in Supplementary Figure 8. Understanding common concepts is vital to designing a good domain-specific language (DSL) to solve tasks that involve these concepts (see Section 3.1).

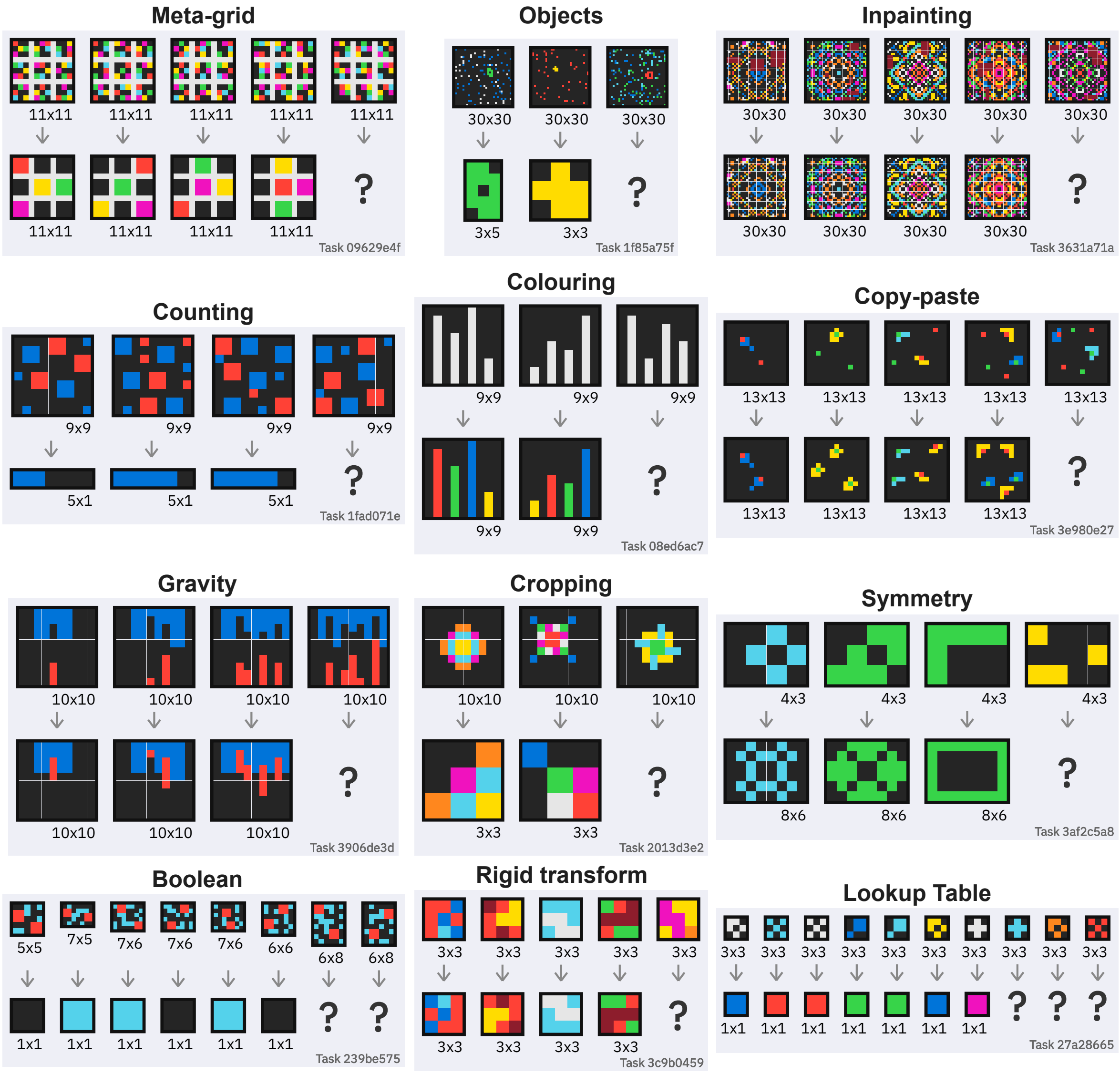

Supplementary Figure 8. Example tasks for 12 taxonomy tags: the diversity of ARC is apparent. Many tasks combine multiple of these concepts or other concepts not shown.

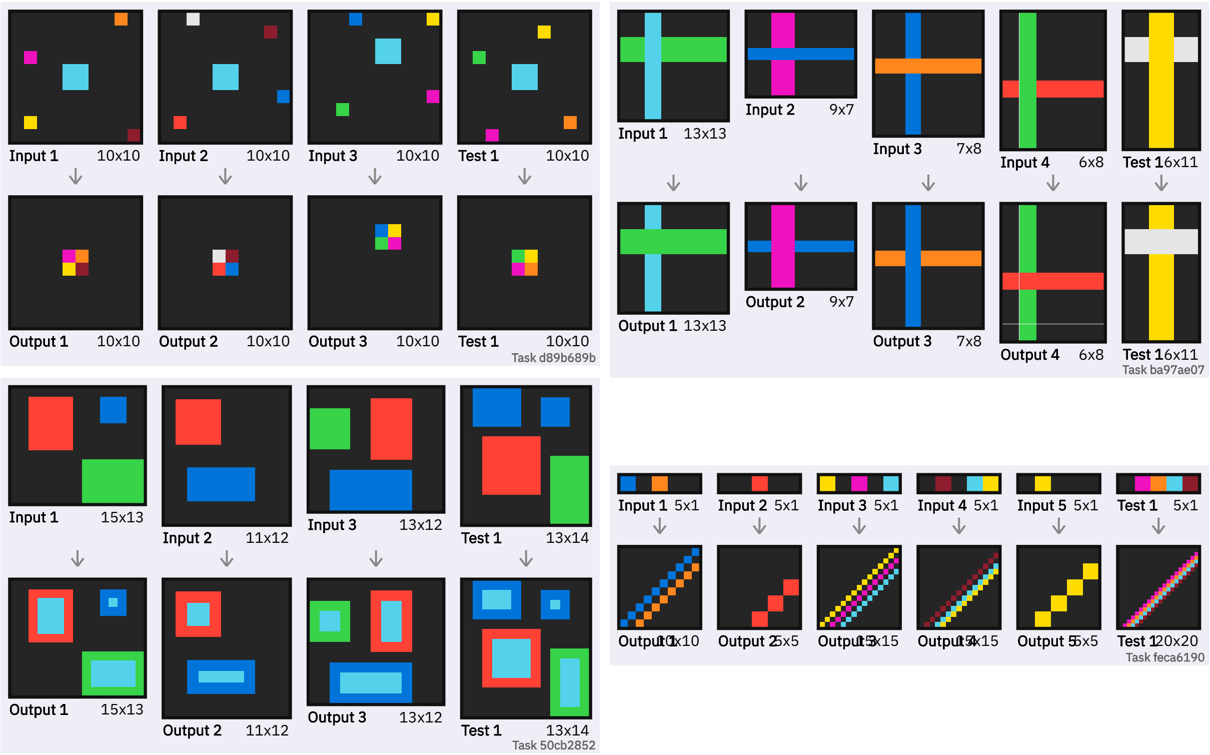

(a) Tasks only solved by Icecuber

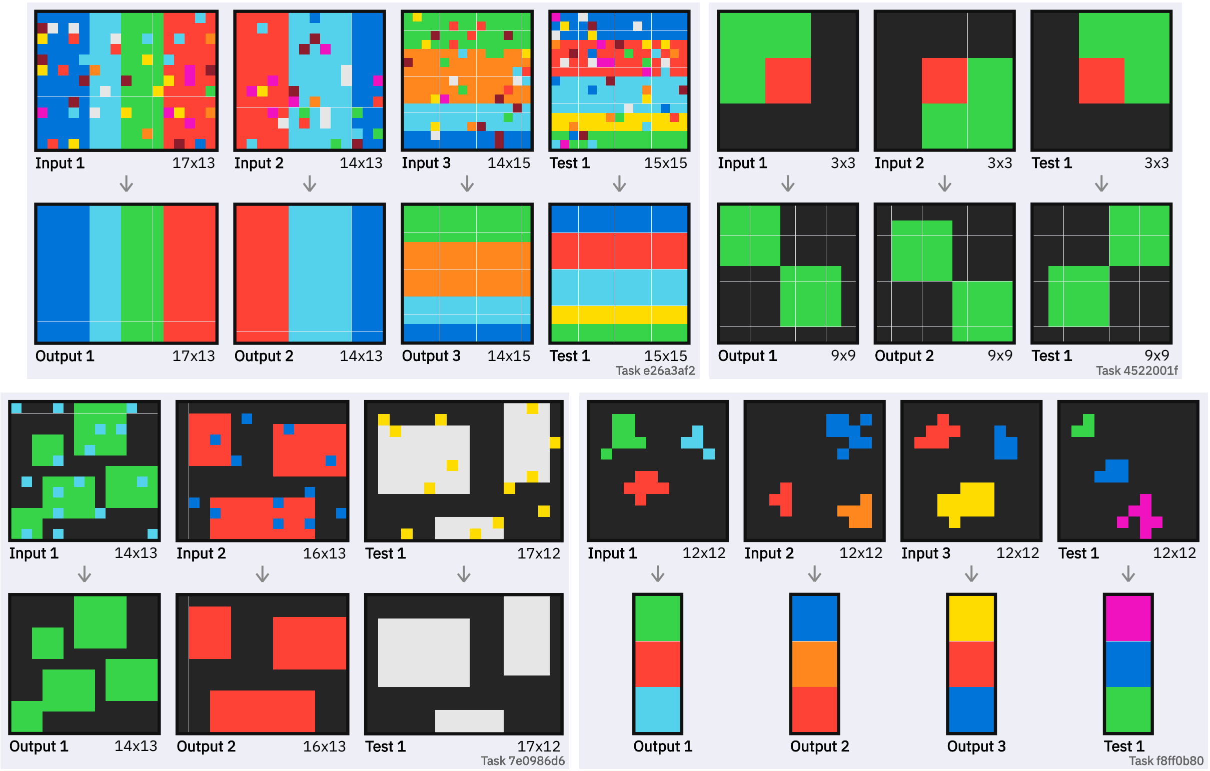

(b) Tasks only solved by GPT-4

Supplementary Figure 7. Randomly selected examples of tasks that only Icecuber, GPT-4 and DreamCoder could solve (respectively). Continued on next page.

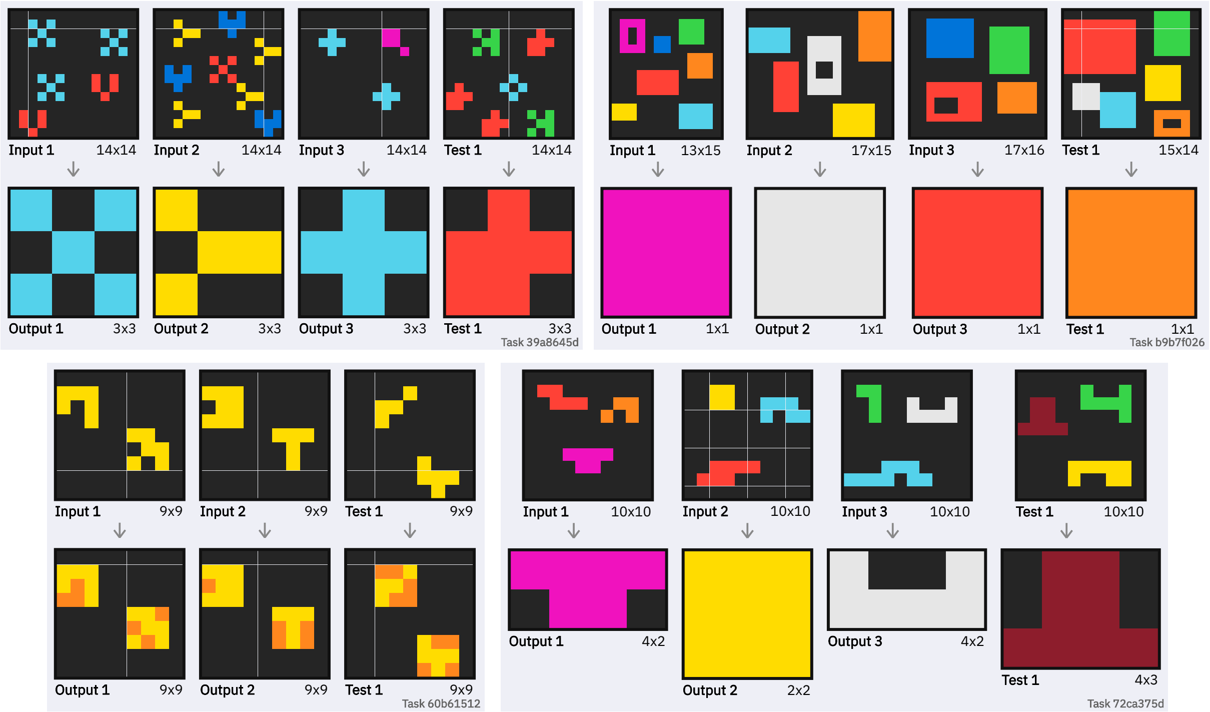

(c) Tasks only solved by DreamCoder. Clockwise from top-left, the programs that DreamCoder wrote to solve the tasks were: `(pickcommon (split8 $0))`, `(ic_compress2 (ic_compress3 (ic_connectX $0)))`, `(pickcommon (ic_splitall (mirrorX $0)))`, `(overlay $0 (mapSplit8 (lambda (set_bg c7 $0)) $0))`

Supplementary Figure 7. Randomly selected examples of tasks that only Icecuber, GPT-4 and DreamCoder could solve (respectively).

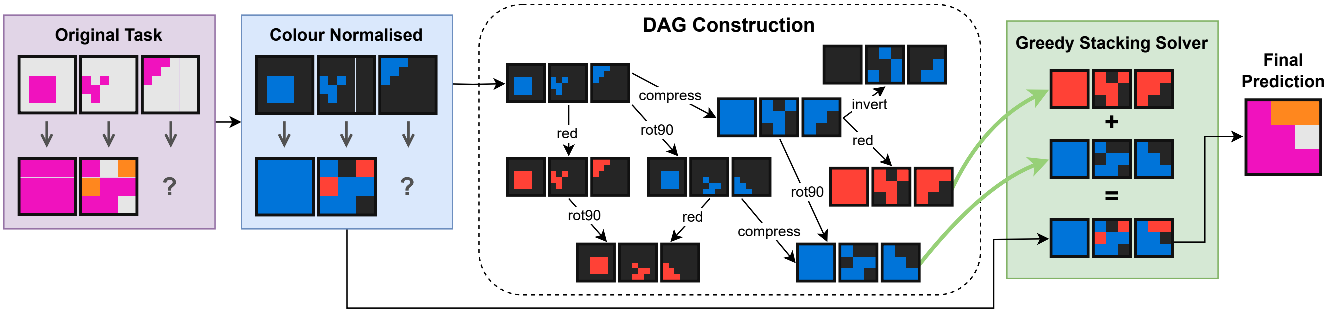

Supplementary Figure 9. Logical diagram of the Icecuber algorithm. Input grids for a task are combined to form 'pieces'. A brute-force search over unary functions on pieces forms a directed acyclic graph (DAG) of potential outputs, until outputs are found that match the task's outputs. If no exact match is found, a greedy stacker combines DAG nodes to produce the final result, minimising pixel distance to the training outputs.

Icecuber: DSL Search

The existing state-of-the-art approach for ARC was introduced by Johan Sokrates Wind (a.k.a **Icecuber**), achieving a 20.6% private test set accuracy [6] in the first Kaggle competition, and has yet to be surpassed.

Icecuber implements a Domain-Specific Language (DSL) in C++, with 42 image transformation functions with 142 total variants [11] (for example, the `eraseColor` function has variants for 10 colours). Each function is a unary transformation from one grid to another, such as cropping, filling in the interior of objects, or re-colouring a grid.

The DSL is combined with a highly efficient brute-force search written in C++, stacking up to 4 unary functions for each task (Supplementary Figure 9). Rather than storing the programs that generate each specific outcome, each function is applied to the entire set of training and test inputs at once (the starting piece), creating a new piece stored in a directed acyclic graph (DAG). After all programs are enumerated, this DAG contains as many as 10^7 pieces (candidate output grids for each input grid). The DAG allows for de-duplication of outputs, which helps with memory usage and performance (*e.g.* `rotate180(rotate180(grid))` points back to the input piece in the DAG, and therefore is not enumerated any further).

Each piece can then be checked against the training data: if all the training inputs are transformed correctly, we have found a candidate solution to our task (and the corresponding test output is already in the piece).

When none of the candidates exactly match the training outputs, the **greedy stacker** can instead be employed. The greedy stacker works by choosing a random subset of training examples, and **selects pieces from the DAG that most closely match the unexplained cells in the grid**, composing them with transparency to minimise the Hamming distance. This allows bidirectional search: finding intermediate states that compose to the output. Finally, solutions are ranked by a complexity heuristic

and the top three solutions are submitted.

Several additional tricks are employed, such as normalising colours in tasks, and a module which guesses the output grid size before enumeration begins.

By limiting the DSL to unary functions only, the search space size can be greatly limited, and becomes amenable to brute-force search. A more complicated DSL that can arbitrarily combine grids would suffer from super-exponential growth, dramatically reducing the maximum depth possible. However, the greedy stacking solver is able to overcome the drawbacks of this by combining grids *after enumeration*, reducing the overall computational complexity.

Prompting LLMs to complete ARC tasks

Tokenisation

For each type of LLM, we use a different grid-encoding scheme to match the tokeniser used by that LLM. An example of how how the LLM 'sees' an ARC problem is shown in Supplementary Figure 10.

LLaMA We encode grids with no spaces between digits, and with newlines between rows. The LLaMA tokeniser creates a separate token per numerical digit.

OpenAI Completion These models use the GPT-2 tokeniser, which has unique tokens for each digit preceded by a space, so we add a space before each cell to maintain one token per grid.

OpenAI Chat These models use the CL100K tokeniser, which **has no unique tokens for digits followed/led by static characters**; therefore, we do not have a way to force each cell to be a single token. We use a tokenisation with no spaces and therefore allow multiple digits to be merged into one token.

```
We are playing a game which involves transformaing a 2D input grid of digits into an output grid of digits. Every below pair
 of grids contains the same transformation (e.g. rotation, symmetry, manipulation of objects). Each Input grid is followed
 by an Output grid which applies the same transformation as previous Input/Output pairs. One such example is below.
Input 1:
 0 0 6
 0 4 0
 3 0 0
Output 1:
 0 0 6
```

Supplementary Figure 10. An example of how the LLM 'sees' an ARC task, with tokenisation highlighted (in this case using OpenAI Completion tokenisation). The task is structured so that each grid cell is a single token, with the test output left as a text completion.

Prompting

Completion prompt format We use the following prompt for completion models:

```
We are playing a game which involves transforming a 2D input grid of digits into an output grid of
digits. Every below pair of grids contains the same transformation (e.g. rotation, symmetry, manipulation
of objects). Each Input grid is followed by an Output grid which applies the same transformation as
previous Input/Output pairs. One such example is below.
```

Chat prompt format Chat models are more complex and use a *system* message followed by interleaved user and assistant messages. We use the following system message:

```
We are playing a game which involves transforming an input grid of digits into an output grid of digits.
In general, digits form objects in 2D and the task is to perform some spatial transformation of these
objects to go from the input grid to the output grid. All the information about the transformation is
contained within the input pairs themselves, and your answer will only be correct if the output grid is
exactly correct, so this is what I expect from you. I will begin by giving you several examples of
input-output pairs. You will then be given a new input grid, and you must provide the corresponding
output grid.
```

We then interleave the input and output grids as User/Assistant messages, so that the chat model can follow the behaviour of what are presented as its previous responses.

DreamCoder Software Architecture

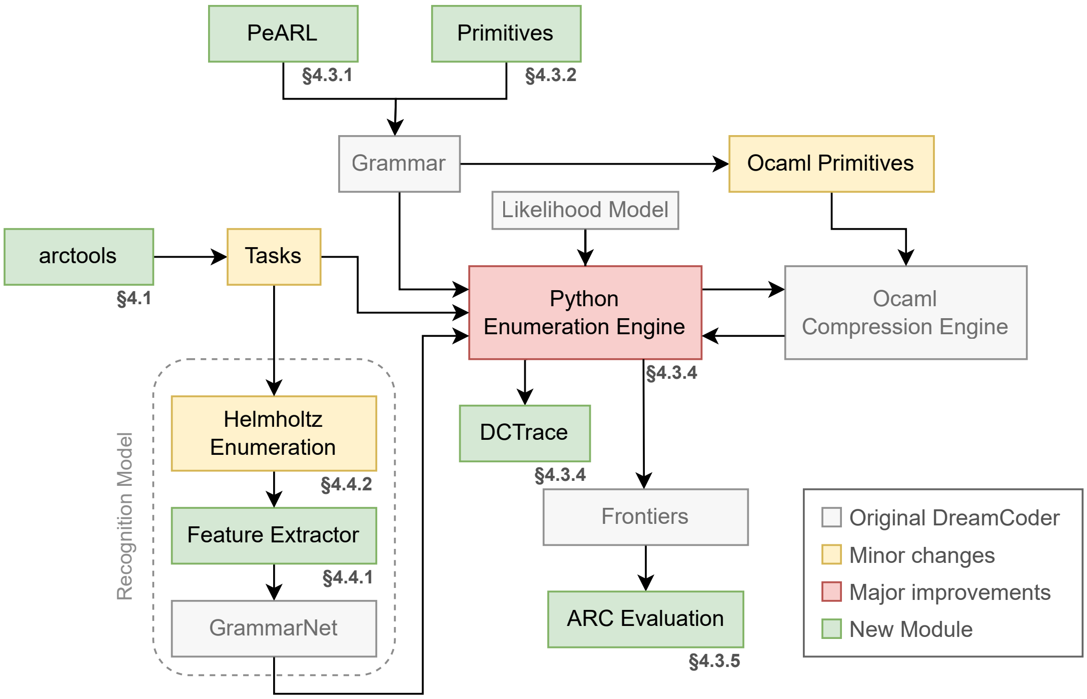

Supplementary Figure 11. An overview of the DreamCoder software architecture, highlighting the primary modules used, and whether they are new or existing modules.

Abstraction Sleep Results

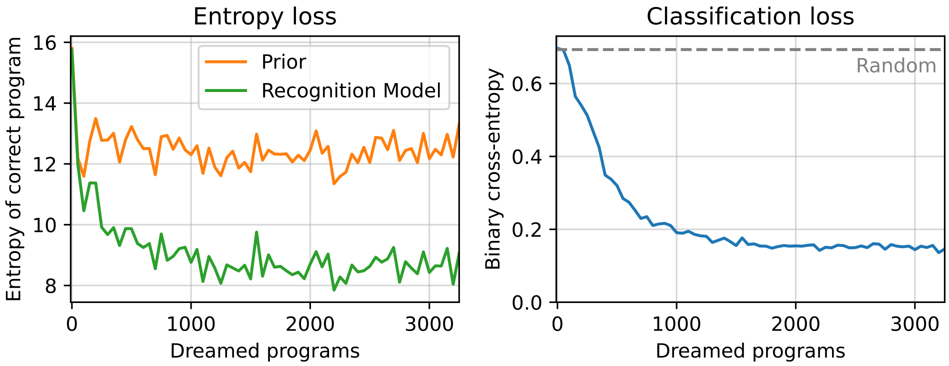

Supplementary Figure 12. Evaluation metrics for the abstraction sleep phase. Entropy loss gives the average entropy of the solutions generated under the neural-network grammar. In addition, we record the binary cross-entropy loss of classifying which primitives are used in a given task. We see that our recognition model is able to guide the search towards the correct solution; this translates to solving more ARC tasks within a given enumeration time limit. A more detailed explanation of these metrics is given below.

Explanation of Entropy loss and Classification loss In Supplementary Figure 12, we provide two metrics. As an illustrative example, suppose that we have three primitives A, B, C and that the correct program is (λ: `B(A($0))`. First, the recognition model produces probabilities of each primitive being contained in the solution $[0.8, 0.7, 0.2]$. The classification loss is the binary cross-entropy of these predictions with $[1, 1, 0]$ (in this case ≈ 0.39).

On the other hand, entropy is defined as $-\log_2(P(p|R))$, where $P(p|R)$ is the likelihood of correct program p being sampled under this recognition model R. This takes into account both the primitives and their configuration ($B(A(\cdot))$ vs. $A(B(\cdot))$), and is the more meaningful metric for actual performance.

So, a 4 bit reduction in entropy means that the correct solution is $2^4 = 16$ times more likely to be sampled during enumeration, and thus that 16 fewer programs need to be sampled to find the solution (although this is on dreamed tasks, the reduction on ARC tasks is approximately 10x). Higher-entropy programs take exponentially longer to find.

PeARL Primitives

The following list provides a complete description of the primitives available in PeARL, along with the number of times they were used in the 88 tasks solved by DreamCoder. A number of these primitives were adapted from the Icecuber C++ DSL [6]. We use `G` to denote the `grid` type.

**Primitive** (Count)	**Type**	**Description**
**Rigid transformations**		
`rot90` (1)	($G \to G$)	Rotate 90° clockwise
`rot180` (6)	($G \to G$)	Rotate 180°
`rot270` (1)	($G \to G$)	Rotate 90° counter-clockwise
`flipx` (3)	($G \to G$)	Horizontal flip
`flipy` (4)	($G \to G$)	Vertical flip
`swapxy` (2)	($G \to G$)	Transpose
**Cropping**		
`left_half` (6)	($G \to G$)	Crop the left half of the grid (floor division)
`right_half` (2)	($G \to G$)	Crop the right half of the grid (floor division)
`top_half` (4)	($G \to G$)	Crop the top half of the grid (floor division)
`bottom_half` (0)	($G \to G$)	Crop the bottom half of the grid (floor division)
**Uncropping**		
`repeatX` (2)	($G \to G$)	Stack two copies of grid horizontally
`repeatY` (0)	($G \to G$)	Stack two copies of grid vertically
`mirrorX` (11)	($G \to G$)	Horizontally mirror grid [abc]->[abccba]
`mirrorY` (11)	($G \to G$)	Vertically mirror grid
`ic_embed` (4)	($G \to G \to G$)	Embeds a grid into a larger hull defined by a 2nd argument (zero-padded)
**Colour manipulation**		
`c[1-9]` (25 total)	(colour)	colour values (9 primitives in total)
`topcol` (6)	($G \to$ colour)	The most common non-black colour
`rarestcol` (5)	($G \to$ colour)	The least common non-black colour
`ic_filtercol` (4)	(colour $\to G \to G$)	Retains only pixels with the specified colour
`ic_erasecol` (8)	(colour $\to G \to G$)	Removes any pixels with the specified colour
`setcol` (7)	(colour $\to G \to G$)	Set all non-black pixels to the specified colour
`set_bg` (7)	(colour $\to G \to G$)	Set black pixels to the specified colour
`get_bg` (1)	(colour $\to G \to G$)	Return grid of background pixels in grid in specified colour
`ic_invert` (2)	($G \to G$)	Replaces black with the `topcol`, replaces colours with black
`colourHull` (1)	(colour $\to G \to G$)	Set every pixel to a colour
**Position manipulation**		
`getpos` (0)	($G \to$ pos)	Get the position of a cropped grid (default 0,0)
`getsize` (0)	($G \to$ size)	Get the size of the grid
`ic_toorigin` (0)	($G \to G$)	Reset a grid's position to $(0, 0)$
**Morphology**		
`fillobj` (5)	(colour $\to G \to G$)	Fill each closed object's interior with a specified colour
`ic_fill` (1)	($G \to G$)	`fillobj` coloured blue
`ic_interior` (0)	($G \to G$)	Return interior of closed objects only, coloured `topcol`
`ic_center` (1)	($G \to G$)	Create a grid of $w/2, h/2$ with centred position, coloured blue
`ic_makeborder` (6)	($G \to G$)	Draw border around objects image in blue (border only)
`ic_spread` (0)	($G \to G$)	Each black cell is coloured with its closest neighbour colour
`ic_spread_minor` (0)	($G \to G$)	`ic_spread` ignoring the most common colour.
**Counting**		
`countPixels` (1)	($G \to$ count)	Return the number of non-black pixels in the input grid
`countcolours` (2)	($G \to$ count)	The number of non-black colours in the input grid
`countComponents` (0)	($G \to$ count)	The number of 4-connected objects in the image
`countToXY` (2)	(count $\to$ colour $\to G$)	Draw a new grid of $n \times n$ with the specified colour
`countToX` (0)	(count $\to$ colour $\to G$)	Draw a new grid of $n \times 1$ with the specified colour
`countToY` (1)	(count $\to$ colour $\to G$)	Draw a new grid of $1 \times n$ with the specified colour
**Compression**		
`ic_compress2` (14)	($G \to G$)	Remove rows/columns which are duplicates of preceeding rows/cols
`ic_compress3` (9)	($G \to G$)	Remove any entirely black rows/columns

Primitive	Type	Description
**Drawing**		
ic_connectX (8)	(G → G)	Join up any objects of the same colour horizontally
ic_connectXY (7)	(G → G)	Connect in both X and Y
**List creation**		
ic_splitcols (1)	(G → list(G))	Split a grid based on colours
ic_splitall (7)	(G → list(G))	Split grid based on 4-connected objects
split8 (4)	(G → list(G))	Split grid based on 8-connected objects
ic_splitcolumns (1)	(G → list(G))	Create $1 \times n$ grids per column
ic_splitrows (0)	(G → list(G))	Create $n \times 1$ grids per row
**List reduction**		
pickcommon (3)	(list(G) → G)	If there are repeated grids, return the most common
ic_pickunique (3)	(list(G) → G)	If there is one unique grid, return it
pickmax_count (0)	(list(G) → G)	Return grid with the most coloured cells
pickmax_neg_count (0)	(list(G) → G)	Return grid with fewest coloured cells
pickmax_size (0)	(list(G) → G)	Return grid with largest area
pickmax_neg_size (0)	(list(G) → G)	Return grid with smallest area
pickmax_cols (3)	(list(G) → G)	Return grid with most colours
pickmax_interior_count (2)	(list(G) → G)	Return the grid with the most empty interior holes
pickmax_neg_interior_count (0)	(list(G) → G)	Return the grid with the fewest empty interior holes
pickmax_x_pos (0)	(list(G) → G)	Return right-most grid
pickmax_x_neg (0)	(list(G) → G)	Return left-most grid
pickmax_y_pos (0)	(list(G) → G)	Return uppermost grid
pickmax_y_neg (0)	(list(G) → G)	Return lowermost grid
**List processing**		
mklist (0)	(G → G → list(G))	Initialise list from two elements
lcons (0)	(G → list(G) → list(G))	List cons
**Composition**		
ic_composegrowing (2)	(list(G) → G)	Overlay grids from largest to smallest, taking into account position
overlay (19)	(G → G → G)	Overlay two grids transparently. If same size, ignore position
logical_and (1)	(G → G → G)	Pixel-wise AND between two grids. Uses colour of first grid
**Higher-order functions**		
mapSplit8 (2)	((G → G) → G → G)	Apply a ((G → G)) lambda to all objects individually
**Gravity**		
gravity_down (2)	(G → G)	Move all objects down with gravity and collisions
gravity_up (0)	(G → G)	Move all objects up with gravity and collisions
gravity_left (0)	(G → G)	Move all objects left with gravity and collisions
gravity_right (1)	(G → G)	Move all objects right with gravity and collisions
